# Machine learning to predict the occurrence of complications after total shoulder arthroplasty for B2-B3 glenoids

**DOI:** 10.3389/fsurg.2025.1637419

**Published:** 2025-10-10

**Authors:** Orlando Parmigiani, Alain Farron, Patrick Goetti, Fabio Becce, Pezhman Eghbali, Alexandre Terrier

**Affiliations:** 1 Division of Orthopaedics and Traumatology, Centre Hospitalier Universitaire Vaudois (CHUV), Lausanne, Switzerland; 2 Department of Diagnostic and Interventional Radiology, Centre Hospitalier Universitaire Vaudois (CHUV), Lausanne, Switzerland; 3 Laboratory of Biomechanical Orthopaedics, Ecole Polytechnique Fédérale de Lausanne (EPFL), Lausanne, Switzerland

**Keywords:** machine learning (ML), reverse total shoulder arthroplasty, anatomical total shoulder arthroplasty, complications, accuracy evaluation, preoperative factors

## Abstract

**Background:**

Total shoulder arthroplasty (TSA) for primary glenohumeral osteoarthritis with B2-B3 glenoids is challenging due to the relatively high rate of postoperative complications, such as glenoid implant loosening. Machine learning (ML) is a promising method for predicting outcomes in shoulder arthroplasty. However, no studies have included preoperative radiological data to predict surgical complications using ML. The present study evaluated the potential of ML in predicting the occurrence of complications after TSA in patients treated for glenohumeral osteoarthritis with B2-B3 glenoids by integrating various prognostic factors, such as radiological features. We hypothesized that ML would accurately predict postoperative complications and identify the variables that are most strongly associated with these complications.

**Materials and methods:**

This retrospective study included 60 patients with primary osteoarthritis and type B2-B3 glenoids from our institutional TSA database. Prognostic factors, including patient characteristics, clinical scores, radiological features, and surgical techniques, were recorded. Outcomes at a minimum of 2 years of follow-up were characterized by the Aldinger complication scale (scored 0-III). Of the 60 patients, 13 (21.7%) experienced complications, with 8 (13.3%) classified as Aldinger I and 5 (8.3%) as Aldinger III. These data were used to train and test four ML methods: logistic regression (LR), gradient boosting classifier (GBC), support vector machine (SVM), and multilayer perceptron classifier (MLPC). We considered a binary outcome: no complication vs. Aldinger I-III. The data were split into a training set (75%) and a testing set (25%).

**Results:**

Among the four ML models evaluated, LR and GBC correctly identified all complication cases (3/12), whereas SVM and MLPC missed one complication. The number of false positives was lower with GBC (2/12) and LR (3/12). Younger age, glenoid version and inclination were the main variables associated with complications. Using a posteriorly augmented glenoid implant was associated with lower complication rates.

**Conclusion:**

ML can efficiently predict TSA complications, even with a limited dataset. Glenoid retroversion was identified as a critical radiological feature associated with outcomes, as supported by the literature. In addition, younger age is associated with increased complication risks, likely due to increased functional demand. Thus, ML is potentially a valuable tool for forecasting complications in the surgical decision-making process.

## Introduction

Managing primary glenohumeral osteoarthritis with posterior glenoid erosion (B1, B2, and B3 according to Walch classification) is challenging, and complications frequently occur (1, 2). Optimal correction of posterior wear should reduce postoperative complications, such as glenoid implant loosening, which is the most common (3–5). Reverse total shoulder arthroplasty (rTSA) is increasingly preferred over hemiarthroplasty and anatomical total shoulder arthroplasty (aTSA) for primary osteoarthritis with posterior glenoid wear. The main biomechanical advantage of rTSA is the semi-constraint of its design, which helps address instability and loosening issues common in aTSA, especially with posterior glenoid erosion (4, 6–10). Previous studies have shown that preoperative factors linked to complications are the American Society of Anesthesiologists (ASA) score, body mass index (BMI), Charlson comorbidity index, humeral head subluxation, and glenoid erosion (11–13).

Machine learning (ML) is increasingly being applied in the medical field to identify patterns within large datasets and support treatment decisions (14). ML is recognized as a valuable tool for predicting complications and outcomes in shoulder arthroplasty. Although ML is promising as an accurate predictive model, a focus on glenoid morphology and on surgical complications is currently lacking. Most studies have focused on clinical scores without integrating radiological variables, which are critical to predicting surgical outcomes (15–19). For example, Gowd et al. demonstrated that ML can be used to successfully predict complications in shoulder arthroplasty, but they did not analyze preoperative images, clinical function, or the type of glenoid wear (18, 20).

The present study aimed to evaluate the potential of ML in predicting the incidence of complications following TSA in patients with primary osteoarthritis and B2-B3 glenoids. The models integrated prognostic factors, including patient characteristics, clinical scores, radiological features, and surgical techniques. According to the literature, the analysis of preoperative radiological features seems to be essential; thus, scapular and glenoid morphology was estimated using a validated deep-learning model (21). This integration of radiological features represents an innovation and a distinctive strength of our study compared to prior score-based approaches. We hypothesized that ML models can accurately predict postoperative complications and identify the variables most strongly associated with these complications despite a limited dataset.

## Materials and methods

### Patient population and data collection

**Figure 1:**
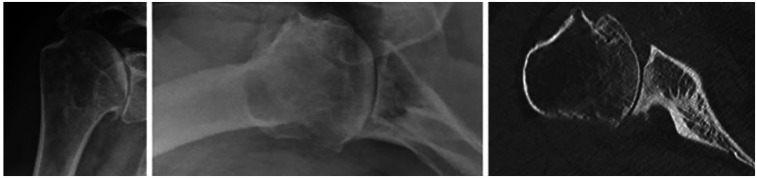
Representative example of shoulder radiographs and CT scan in a patient with primary glenohumeral osteoarthritis and B2 glenoid wear.

We used our institutional database of TSA procedures for this retrospective single-center study and included patients with a minimum radiological and clinical follow-up of 2 years after rTSA or aTSA for primary glenohumeral osteoarthritis with posterior glenoid wear classified as B2 or B3 according to Walch. To assess preoperative radiological features, all patients must have undergone a preoperative shoulder CT scan (Figure 1). Sixty patients met the inclusion criteria and were included in the study (Table 1).

**Table 1:** Preoperative variables and occurrence of complication.

Characteristic	Category	Grouped by Aldinger				
		Missing	Overall	0	1	3
*n*			60	47	8	5
Age, mean (SD)		0	71.7 (8.3)	73.2 (8.1)	66.9 (4.7)	65.7 (10.8)
Sex, *n* (%)	F	0	36 (60.0)	30 (63.8)	4 (50.0)	2 (40.0)
	M		24 (40.0)	17 (36.2)	4 (50.0)	3 (60.0)
BMI, mean (SD)		0	28.6 (5.4)	28.8 (4.9)	27.7 (6.5)	28.2 (9.1)
ASA, *n* (%)	1.0	0	4 (6.7)	4 (8.5)		
	2.0		35 (58.3)	26 (55.3)	6 (75.0)	3 (60.0)
	3.0		21 (35.0)	17 (36.2)	2 (25.0)	2 (40.0)
Tobacco, *n* (%)	N	0	54 (90.0)	41 (87.2)	8 (100.0)	5 (100.0)
	Y		6 (10.0)	6 (12.8)		
Alcohol, *n* (%)	N	0	49 (81.7)	39 (83.0)	6 (75.0)	4 (80.0)
	Y		11 (18.3)	8 (17.0)	2 (25.0)	1 (20.0)
Comorbidities, mean (SD)		0	20.7 (11.6)	22.2 (12.1)	15.2 (7.1)	15.7 (9.3)
Handedness *n* (%)	L	0	24 (40.0)	19 (40.4)	4 (50.0)	1 (20.0)
	R		36 (60.0)	28 (59.6)	4 (50.0)	4 (80.0)
Walch, *n* (%)	B2	0	36 (60.0)	26 (55.3)	7 (87.5)	3 (60.0)
	B3		24 (40.0)	21 (44.7)	1 (12.5)	2 (40.0)
Version, mean (SD)		0	−10.7 (10.4)	−9.7 (9.4)	−18.7 (9.8)	−7.7 (16.2)
Inclination, mean (SD)		0	5.5 (6.5)	4.7 (6.8)	7.6 (5.3)	9.0 (3.7)
Subluxation, mean (SD)		0	−17.4 (13.1)	−17.4 (12.9)	−19.9 (10.7)	−13.0 (19.2)
Glenoid density, mean (SD)		0	495.1 (154.6)	476.5 (145.6)	530.3 (71.3)	614.4 (275.1)
Abduction, mean (SD)		2	86.7 (22.1)	86.0 (23.0)	85.7 (16.2)	97.5 (22.2)
Flexion, mean (SD)		2	95.1 (21.2)	94.4 (22.6)	94.3 (11.3)	105.0 (17.3)
External rotation, mean (SD)		2	9.2 (10.4)	9.5 (10.6)	8.6 (10.7)	7.5 (9.6)
Internal rotation (according to constant, 1–6), mean (SD)		2	3.5 (1.7)	3.4 (1.7)	3.4 (1.5)	4.5 (1.9)
Treatment, *n* (%)	aTSA	0	23 (38.3)	14 (29.8)	6 (75.0)	3 (60.0)
	rTSA		37 (61.7)	33 (70.2)	2 (25.0)	2 (40.0)
Planning, *n* (%)	N	0	20 (33.3)	12 (25.5)	4 (50.0)	4 (80.0)
	Y		40 (66.7)	35 (74.5)	4 (50.0)	1 (20.0)
PSI guide, *n* (%)	N	0	23 (38.3)	15 (31.9)	4 (50.0)	4 (80.0)
	Y		37 (61.7)	32 (68.1)	4 (50.0)	1 (20.0)
Graft, *n* (%)	Augmented	0	30 (50.0)	27 (57.4)	2 (25.0)	1 (20.0)
	Graft		7 (11.7)	4 (8.5)	1 (12.5)	2 (40.0)

The surgeries were performed by a single experienced surgeon between 2012 and July 2020. Patients were assessed clinically and radiologically prior to surgery and postoperatively at 6 weeks, 3 months, 6 months, 24 months, and at last available follow-up. Preoperative radiological data were extracted from the CT scan, whereas postoperative radiological assessments consisted of standard anteroposterior shoulder radiographs, lateral/scapular Y views, and superior-inferior axial views. Patient-related preoperative variables included age, sex, BMI, tobacco and alcohol consumption, number of medications, shoulder dominance, ASA score, and preoperative clinical scores from the American Shoulder and Elbow Surgeons (ASES) and Simple Shoulder Test (SST). The four radiological variables (glenoid version, glenoid inclination, humeral head subluxation, and glenoid bone density) were measured in 3D from the preoperative CT scan for each patient. The glenoid density was measured in the subchondral trabecular bone region (22–25). The bone segmentation and anatomical landmarking were obtained by using a deep-learning model (21). These CT measurements were obtained automatically for each patient.

Perioperative and surgical variables were also analyzed, including the type of prosthesis used (rTSA vs. aTSA), the technique used to correct posterior glenoid wear (ream and run technique, bone graft, or augmented implant), the use of 3D planning, and whether a patient-specific instrument (PSI) cutting guide was used.

The surgical outcome was reported as the Aldinger score (0-III), a system for categorizing complications that has been found to be applicable for standardizing complications in shoulder arthroplasty (Table 2), (26). Arthroplasty complications are categorized into three levels, focusing on the severity and impact on treatment. Complications range from issues requiring no surgical intervention to complications requiring soft tissue revision and cases requiring implant revision. This system provides a clear and standardized approach for assessing and managing different types of complications following shoulder arthroplasty.

**Table 2:** Methodology of classification according to Aldinger.

Category	Description
I	Complications not requiring intervention: Nerve injury (transient or permanent) Periprosthetic radiolucency/implant loosening, asymptomatic and without surgical revision
II	Complications requiring soft tissue revision: Surgical revision for soft tissue infection without implant exchange
III	Complications requiring implant revision: Infection necessitating revision of prosthetic implants Symptomatic loosening Dislocation

For patients who received a PSI cutting guide (*n* = 37, 61.7%), it was 3D printed based on the morphology of the glenoid on CT. The deltopectoral approach was used. All implants, for both aTSA and rTSA, were from Tornier (Stryker). In addition, the Aequalis Ascend Flex stem was used. Regarding the glenoid components, the Perform + glenoid was used for aTSA and the Perform reverse and Perform reverse augmented were used for rTSA.

Among the 60 patients included in this study, 36 (60%) were female and 24 (40%) were male. The mean patient age was 71.7 years, and the mean follow-up was 76.2 months. Twenty-three patients (38.3%) and 37 patients (61.7%) underwent aTSA and rTSA, respectively. Our series included 36 glenoids classified as B2 and 24 as B3 according to Walch (Table 1).

### Machine learning models

Data pre-processing, development and tuning of the ML models, and post-processing of the results were coded in Python, mainly using the scikit-learn libraries (scikit-learn.org). We split the data as 75% for training and validation and 25% for testing with the function train_test_split of scikit-learn, stratifying by Aldinger class. As our data contain a few missing data, we used the function IterativeImputer of scikit-learn to impute these missing values (Table 1). We binarized the four levels of Aldinger complications as “false” for an Aldinger value of 0 and “true” for Aldinger values of I, II, or III. We used the function SMOTENC from the imblearn Python library (imbalanced-learn.org) to balance, and extend, the number of samples (i.e., patients) in the two complication groups of the training dataset. We conducted missing data imputation and SMOTENC balancing exclusively within the training folds during cross-validation to prevent data leakage into the test sets and to ensure the validity of the model assessment. Thus, we had 35 patients per group in the training dataset, 70 in total. In the untouched testing dataset, 12 patients did not have complications and 3 had complications.

We used four standard ML models to predict the binary complication: logistic regression (LR), gradient boosting classifier (GBC), support vector machine (SVM), and multilayer perceptron classifier (MLPC). For the four models, we used the same techniques as described above to prepare the data. We used the GridSearchCV algorithm from scikit-learn to optimize (fine-tune) the hyperparameters of the four ML models. We optimized the F1 score and used 5-fold cross-validation for optimization of these hyperparameters, without repeated or nested cross-validation.

The performance of the four methods was evaluated in the same way with the untouched test dataset using precision (i.e., percentage of true positives), recall (i.e., percentage of true positives out of all actual positive cases), F1 score (i.e., harmonic mean of precision and recall), accuracy (i.e., percentage of correct predictions out of all predictions, 95% CI), and area under the receiver operating characteristic (ROC) curve (AUC). We also reported the confusion matrix as well as prevalence-aware metrics (PR-AUC, balanced accuracy) and calibration measures (Brier score).

## Results

### Complications

A total of 13 complications were identified (21.7%) among the 60 included patients. Patients who underwent aTSA were associated with a higher incidence and severity of complications than rTSA patients. Specifically, nine complications were observed among the 23 patients who underwent aTSA (39.1%) and four complications among the 37 patients who underwent rTSA (10.8%). Eight patients (13.3%) had a class I complication, six of which underwent aTSA and two rTSA. Five patients (8.3%) had a class III complication, three of which underwent aTSA and two rTSA (Table 1). No patients had class II complications.

Aldinger I complications occurred in six patients with radiological glenoid implant loosening, one patient with humeral stem loosening, and a nerve injury comprising transient hypoesthesia in the ulnar nerve territory. Aldinger III complications occurred in three patients with aseptic glenoid implant loosening, one patient with chronic infection with glenoid implant loosening, and one patient with stem loosening associated with a periprosthetic fracture. Two patients with aseptic loosening had conversion to rTSA, and one patient received a hemiprosthesis due to insufficient glenoid bone stock. The patient with chronic infection received a cement spacer but was not reimplanted due to cardiac issues. Finally, the patient with stem loosening and fracture underwent open reduction and internal fixation with rTSA.

**Figure 2:**
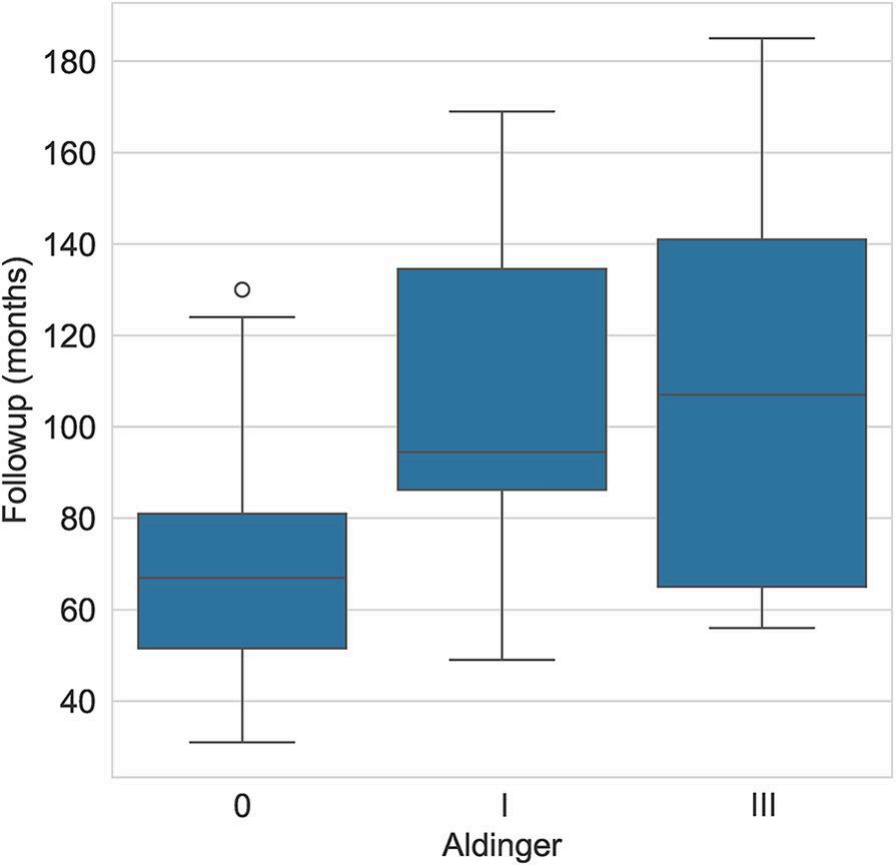
Aldinger complications by follow-up (months).

The average (± standard deviation) glenoid inclination for all study patients was 5.5° (± 6.5°). The average inclination was 7.6° (± 5.3°) for patients with Aldinger type I complications and 9° (± 3.7°) for those with type III complications. The mean glenoid version was −10.7° (± 10.4°) and the mean subluxation index was −17.4 (± 13.1). For patients with type I complications, the mean version was −18.7° (± 9.8) and −19.9° (± 10.7), respectively for glenoid version and subluxation index. For patients with type III complications, the mean was −7.7° (± 16.2) and −13° (± 19.2), respectively. The mean follow-up for patients who experienced an Aldinger I complication was 107.2 months and 110.8 for Aldinger II patients, whereas the mean follow-up was 67.3 months for those without complications (Figure 2).

**Figure 3:**
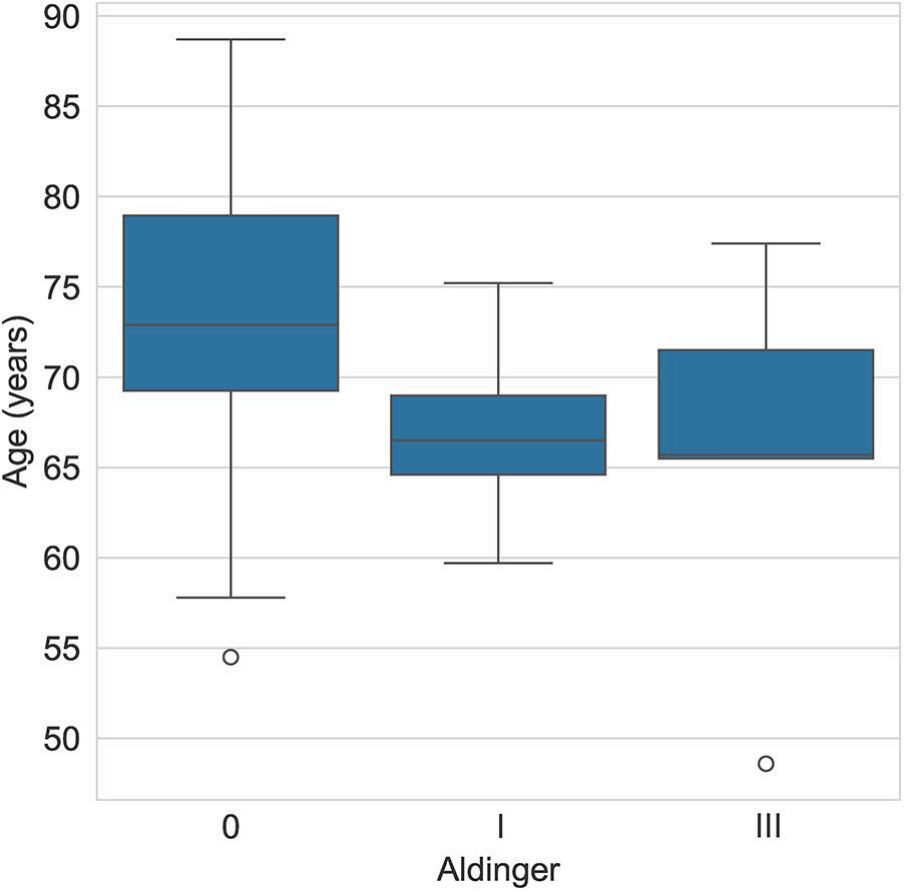
Aldinger complications by age (years).

The mean age of patients who experienced a type I complication was 66.9 years (± 4.7) and 65.7 years (± 10.8) for those with a type III complication, whereas it was 73.2 years (± 8.3) for those without complications (Table 1 and Figure 3).

### Machine learning prediction

**Figure 4:**
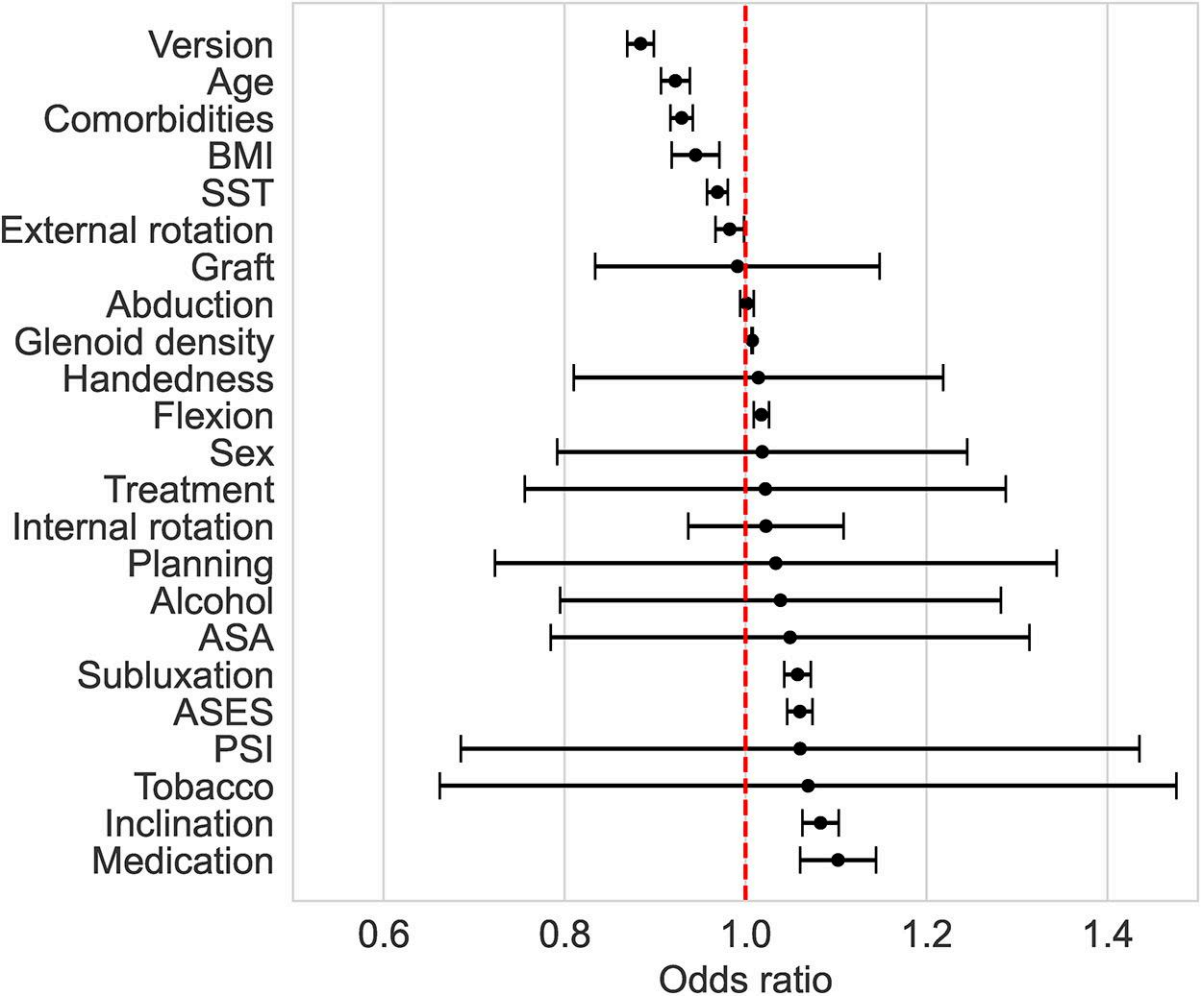
Odds ratio (with 95% confidence interval) associated with the preoperative factors of the LR method. An odds ratio >1 means that an increase in the predictive variable is associated with higher odds of complications.

**Figure 5:**
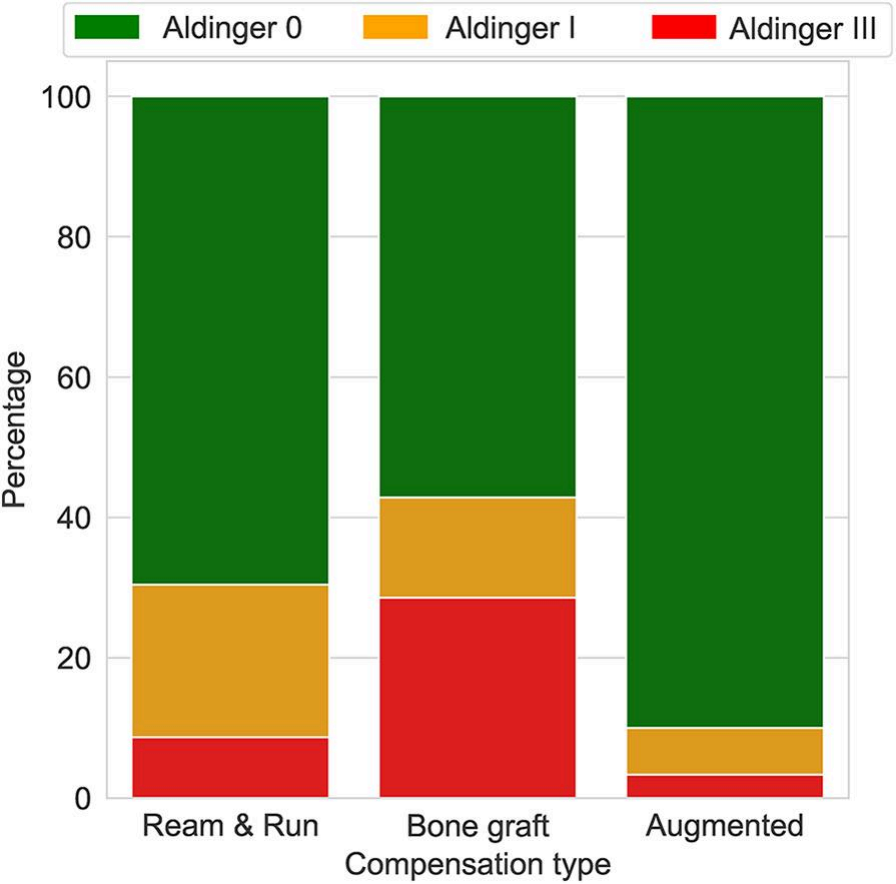
Complication occurrence depending on posterior wear correction technique (percentage).

Only GBC and LR correctly predicted the three cases of complications (Table 3). SVM and MLPC wrongly predicted one of the three cases with complication as without complication. The number of false positives was lowest with GBC (2/12), followed by LR (3/12) and SVM (3/12), and then MLPC (4/12). According to the LR and GBC models, younger age, good preoperative general health (e.g., fewer comorbidities, lower BMI, and higher preoperative ASES score), and glenoid version and inclination were the main variables predicting complications (Figure 4). Using a posteriorly augmented glenoid implant was associated with lower complication rates (Figure 5).

**Table 3:** Performance of the 4 ML methods (train and test): logistic regression (LR), gradient boosting classifier (GBC), support vector machine (SVM), multi-layer perceptron classifier (MLPC).

Metric	LR		GBC		SVM		MLPC	
	Train	Test	Train	Test	Train	Test	Train	Test
Class count	35	12	35	12	35	12	35	12
Confusion matrix	[[30 5] [ 1 34]]	[[9 3] [0 3]]	[[33 2] [ 0 35]]	[[10 2] [ 0 3]]	[[35 0] [ 3 32]]	[[9 3] [1 2]]	[[35 0] [ 0 35]]	[[8 4] [1 2]]
Precision	0.87	0.50	0.95	0.60	1.00	0.40	1.00	0.33
Recall	0.97	1.00	1.00	1.00	0.91	0.67	1.00	0.67
F1	0.82	0.67	0.97	0.75	0.96	0.50	1.00	0.44
Accuracy	0.91	0.80	0.97	0.87	0.96	0.73	1.00	0.67
95% CI	[0.84, 0.97]	[0.60, 1.00]	[0.93, 1.00]	[0.67, 1.00]	[0.90, 1.00]	[0.47, 0.93]	[1.00, 1.00]	[0.40, 0.87]
Balanced accuracy	0.71	0.88	0.97	0.92	0.96	0.71	1.00	0.67
AUC	0.936	0.833	0.971	0.916	0.999	0.861	1.00	0.806
95% CI	[0.851, 0.999]	[0.583, 1.000]	[0.929, 1.000]	[0.800, 1.000]	[0.995, 1.000]	[0.483, 1.000]	[1.000, 1.000]	[0.375, 1.000]
PR-AUC	0.777	0.372	0.999	0.656	0.999	0.777	1.000	0.754
Brier score	0.177	0.187	0.065	0.148	0.136	0.150	0.000	0.326

## Discussion

The results of this study demonstrate that ML, even using only 60 patients, can be accurate and reliable in predicting complications after aTSA or rTSA. While LR and GBC correctly identified all complication cases, SVM and MLPC each missed one complication. This supports the point that LR seems sufficient to predict complications (i.e., the risk) after TSA. Nevertheless, the reported false positive rate of 16.7% is too high to rely on ML to identify whether a specific patient is eligible for shoulder arthroplasty.

All 23 input variables were useful in predicting the occurrence of complications, but some of them had a stronger association with the outcome. This can be seen from the odds ratio of the LR, as well as from the feature importance of the GBC.

**Figure 6:**
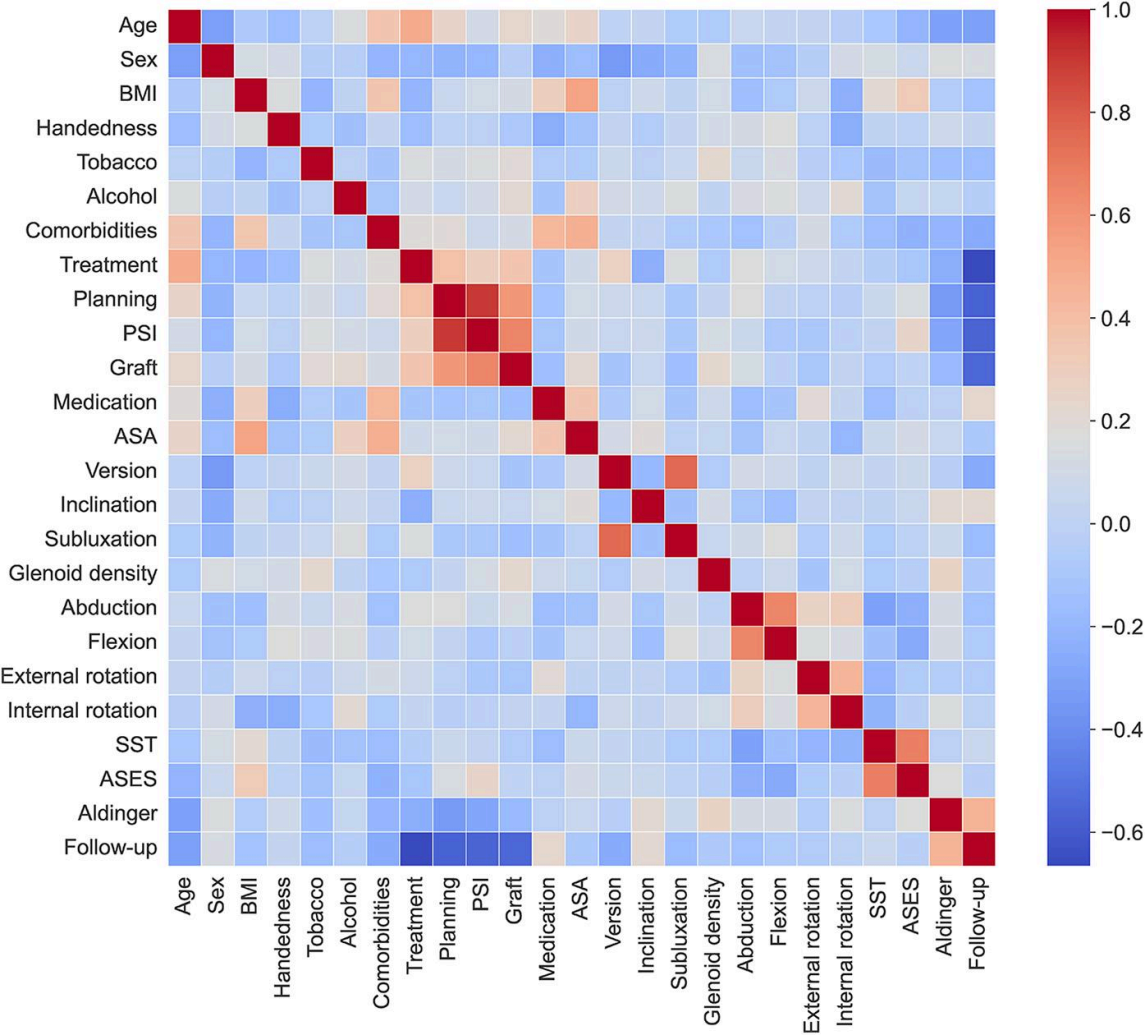
Pearson correlation matrix of the collected data.

As comorbidities, BMI, and preoperative ASES correlate with age, we can reasonably assume that younger patients are more likely to experience complications because they have higher functional demand (Figure 6). This observation is in line with the results and comments by Farng et al. (27). We assumed that patients in good preoperative health (e.g., younger, less medication) are more likely to experience complications because they have higher functional demand and put greater stress on their glenohumeral joint. Furthermore, younger patients, due to their longer follow-up, are more likely to develop complications over time. Regarding humeral head subluxation, the results of our study correlate with the findings of Lee et al. and Walch et al. (13, 28). Indeed, Lee et al. found that patients with posterior humeral head subluxation had lower ASES scores, increased pain, and decreased active external rotation. Walch et al. demonstrated that humeral head subluxation was significantly associated with an increased risk of prosthetic dislocation and glenoid component loosening.

Although aTSA appears to have a higher complication rate, the ML approach employed in this study is purely predictive. Consequently, the challenge in interpreting why the treatment variable (aTSA/rTSA) demonstrates an odds ratio near 1 with a wide confidence interval stems from the presence of numerous correlated variables in the model, which likely impact the regression outcomes (Figure 6).

Previous ML studies on shoulder arthroplasty mostly evaluated clinical outcome and score predictions and, when examining complications, included medical issues and any causes of readmission, but none focused on the type of glenoid wear and preoperative radiological assessment (15, 16, 29, 30). Kumar et al. demonstrated that ML can be used in shoulder arthroplasty, even with a reduced dataset as in the present study (19). Devana et al. found that XGBoost was the best model for predicting the occurrence of complications, and prior history of complications and chronic kidney disease were the variables most associated with the occurrence of complications (30). Lopez et al. studied the use of ML to predict the risk of non-home discharge and found that an ASA classification of 3 or greater and history of diabetes were the main predictive variables (31).

These results differ from those of our study, probably because the outcomes and studied data were different, as they took into account all medical complications. In our study, we defined complications using the Aldinger system and focused on surgical complications.

A key strength of this study is the inclusion of radiological features in the ML models and, more specifically, using a deep-learning model for automatic measurements of CT scans for each patient to predict complications after shoulder arthroplasty. By including these radiological features alongside clinical variables, the study enhances the model's predictive accuracy, particularly in cases of posterior glenoid wear. To the best of our knowledge, this is the first study including such features. In addition, the model's use of 23 variables, including patient comorbidities and clinical scores, enabled a broad range of predictors for complications to be captured.

The study is limited by the small sample size due to low patient volume and, consequently, low number of complications. The retrospective design and exclusive focus on B2 and B3 glenoid types contributed to the constraints on the dataset. Thus, we had to binarize the Aldinger complication categories, predicting any complication. Extending the number of patients for training the ML reduced the false positive rate and better identified the relative importance of the variables associated with complications.

The important findings of our study are predictive and not causal. The ML models we employed aim to forecast the likelihood of complications based on a range of clinical and radiological features rather than establishing direct cause-and-effect relationships between specific variables and outcomes. Nevertheless, ML allowed us to identify patterns and predictors of complications.

We think that predictive models trained with an artificially-balanced dataset cannot be used directly in a clinical setting. For such a use, further research with expanded patient numbers will allow better validation, ensuring more robust clinical applications.

## Conclusion

ML can predict complications of TSA, even with a limited dataset. Our study confirms that glenoid retroversion and inclination, as reported previously in the literature, may be critical determinants influencing outcomes. Therefore, preoperative scapular and glenoid morphology is an important variable to quantify and should be considered when using ML models for predicting postoperative complications. Furthermore, younger age is linked to higher complication risks, likely due to increased functional demand. Further studies with more patients are required to validate our model, but the use of ML for shoulder arthroplasty offers promising avenues for improving surgical outcomes. These technologies may help predict postoperative complications, identify high-risk patients, and optimize preoperative planning, contributing to more personalized and effective patient care.

## Data Availability

The raw data supporting the conclusions of this article are available on Zenodo https://doi.org/10.5281/zenodo.17203516. Further inquiries can be directed to the corresponding author.
